# Supplementary figures and images for: Characterizations of the viability and gene expression of dispersal cells from Pseudomonas aeruginosa biofilms released by alginate lyase and tobramycin
Source: PLoS One. 2021 Oct 25;16(10):e0258950. doi: 10.1371/journal.pone.0258950 (PMC8544826; doi:10.1371/journal.pone.0258950)

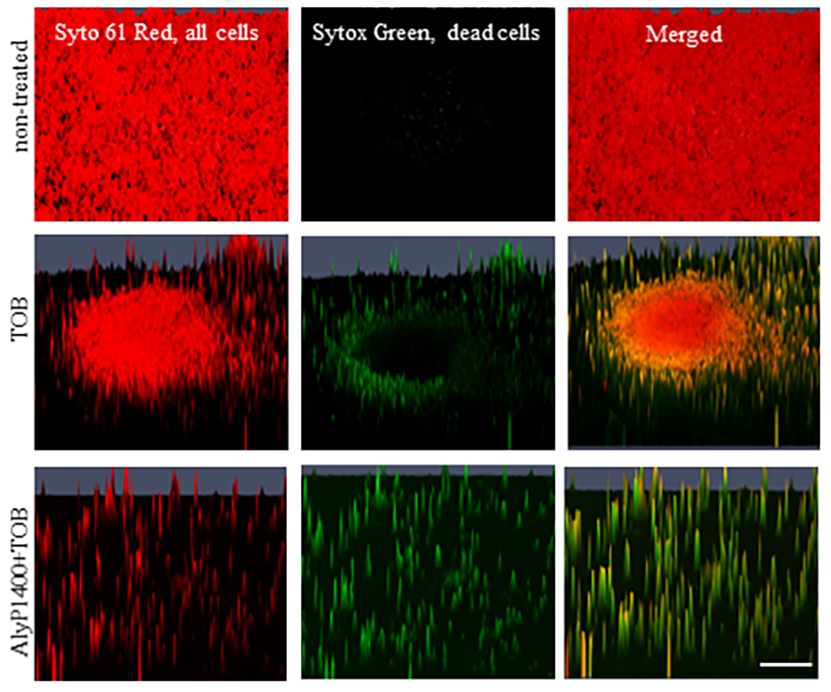

Supplement: S1 Fig — The residual biofilms were visualized with Confocal Laser Scanning Microscopy (CLSM) after staining with Syto 61 Red, (stained all cells red) and Sytox Green, (stained compromised membrane and /or dead cells green) using magnification power 63X. The yellow fluorescence represented merging of both red and green channels in the merged panel. Images are representatives from three independent replicates with 20 μm bars. (TIF) [file pone.0258950.s001.tif]

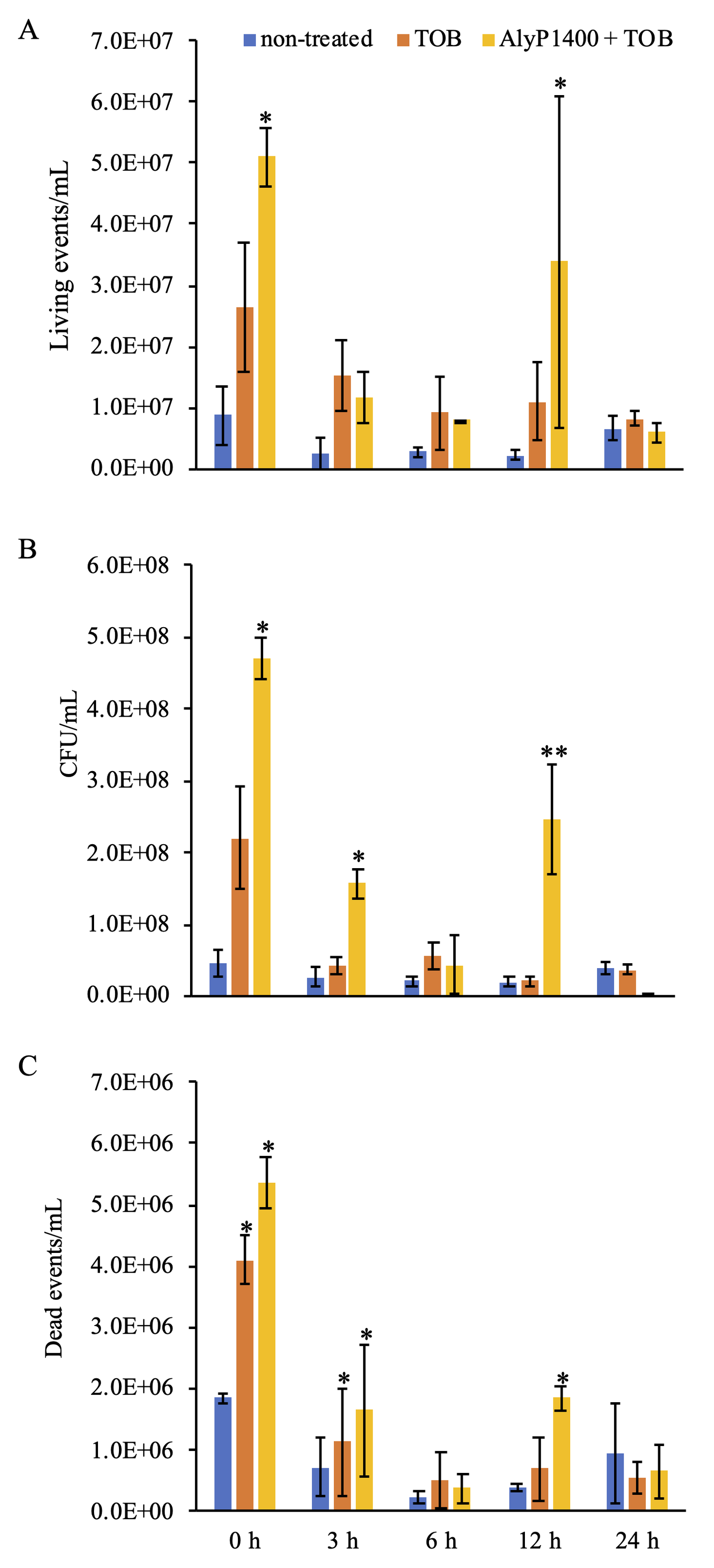

Supplement: S2 Fig — Sequential treatment (AlyP1400→TOB) data were only acquired for 0 h time point, therefore were not included in this figure. (A) Live events per millilitre (events/mL) as determined using flow cytometric analysis of the cell populations for living cells (events negative for FVS520 and positive for FVS700). (B) The total viable count (CFU/mL) of P. aeruginosa CF27 living dispersed biofilm cells. (C) Dead events per millilitre (events/mL) as determined using flow cytometric analysis of the dead populations (events positive for both FVS520 and FVS700). Statistical significance was determined using ANOVA two-way analysis with Tukey’s test multiple comparison test. * P < 0.05, ** P < 0.01 compared with non-treated control. The standard error of mean of three independent flow cells is indicated by the error bars. (TIF) [file pone.0258950.s002.tif]

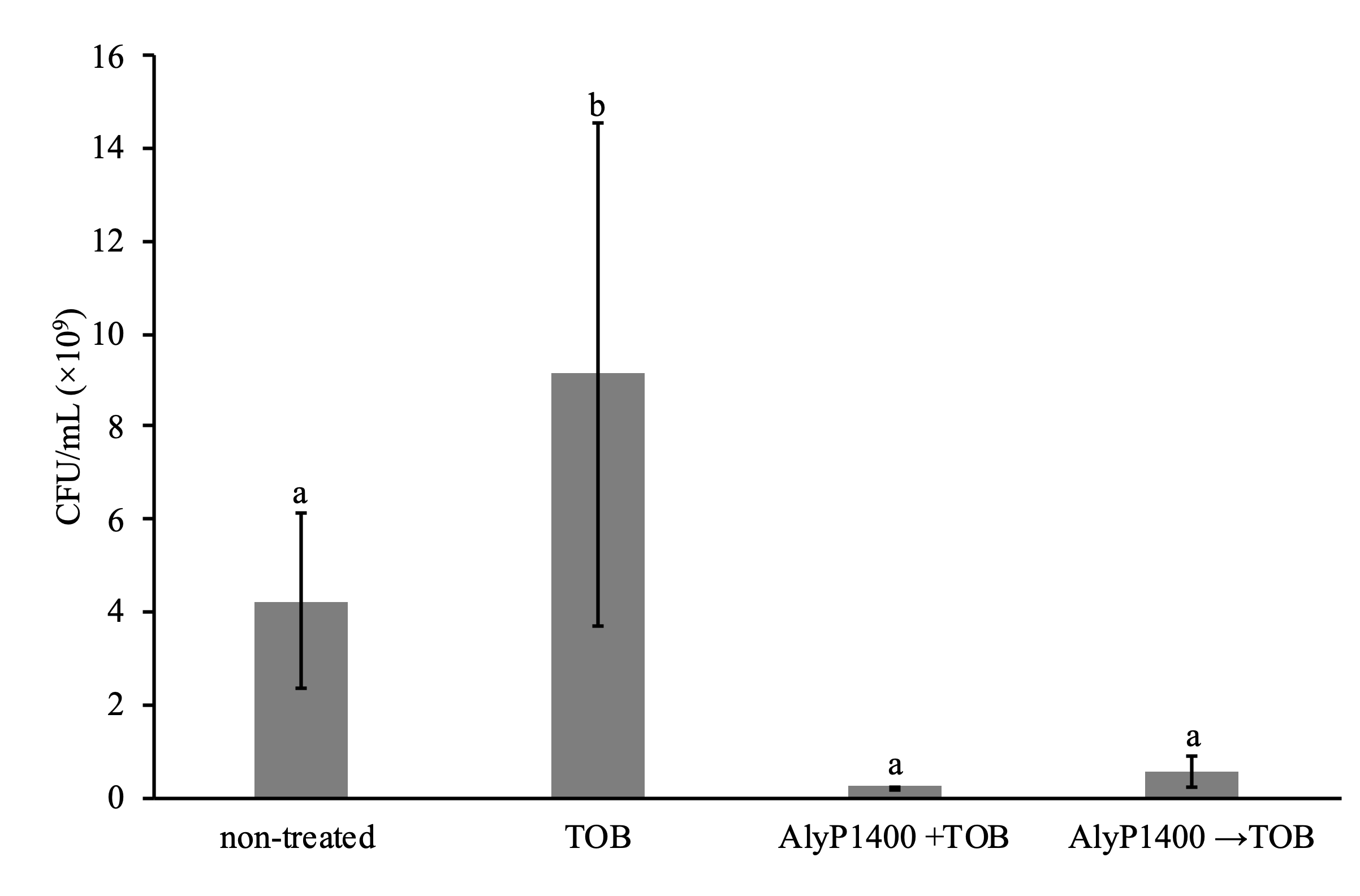

Supplement: S3 Fig — Bars with different letters (a and b) are statistically different (p<0.05, ANOVA two-way with Tukey’s multiple comparison test). Data were analyzed for samples collected immediately after the treatments. The standard error of mean of three independent flow cells is indicated by the error bars. (TIF) [file pone.0258950.s003.tif]

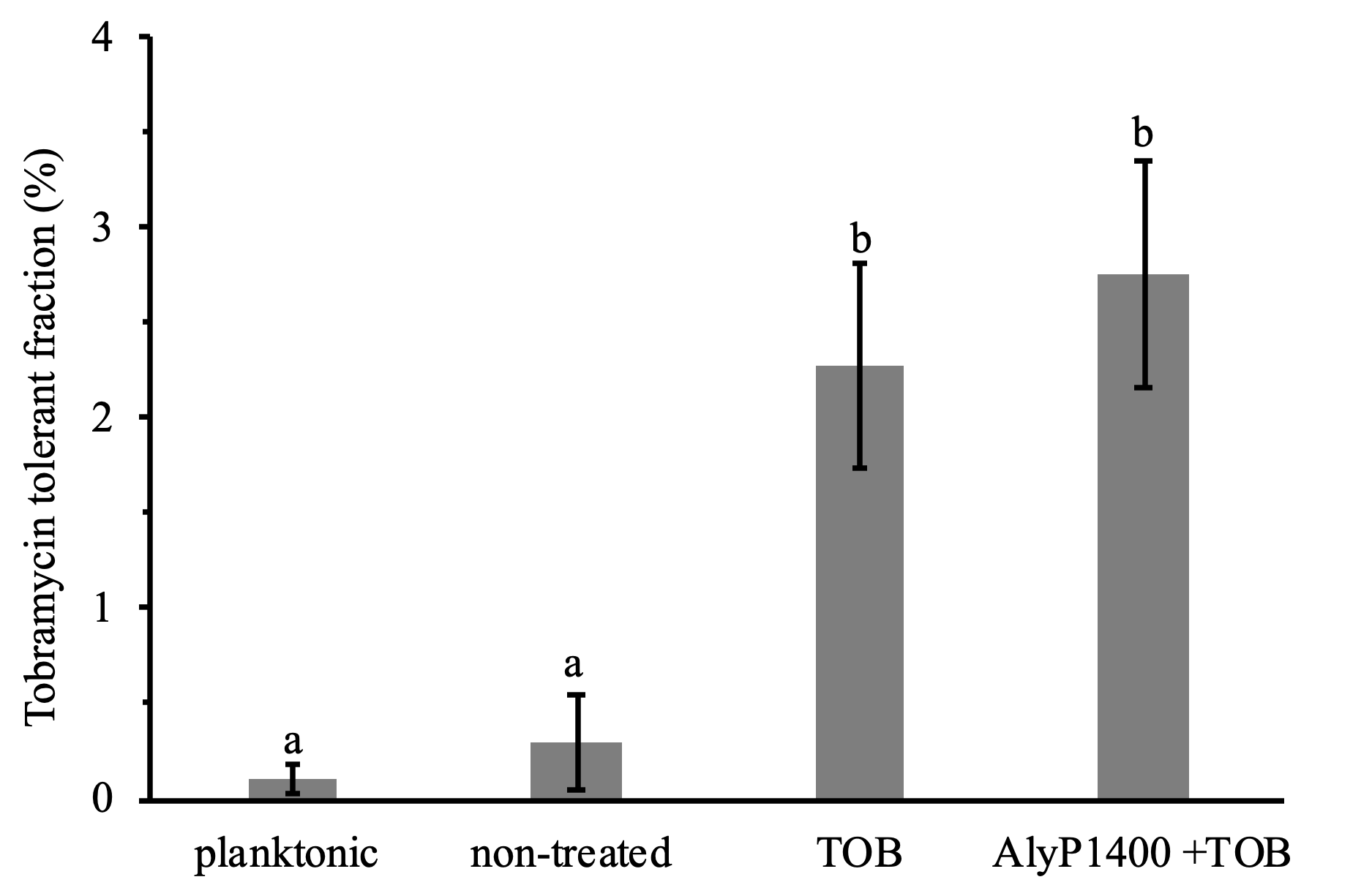

Supplement: S4 Fig — Bars with different letters (a and b) are statistically different (p<0.05, ANOVA one-way with Tukey’s multiple comparison test). Data were analyzed for samples collected immediately after a 2-h treatment with buffer control (non-treated), or 16 μg/mL tobramycin (TOB) or with 250 U/mL AlyP1400 and 16 μg/mL tobramycin (AlyP1400+TOB). The standard error of mean of three independent flow cells is indicated by the error bars. (TIF) [file pone.0258950.s004.tif]
