# Supplementary material for: Characterizations of the viability and gene expression of dispersal cells from Pseudomonas aeruginosa biofilms released by alginate lyase and tobramycin
Source: PLoS One. 2021 Oct 25;16(10):e0258950. doi: 10.1371/journal.pone.0258950 (PMC8544826; doi:10.1371/journal.pone.0258950)
Supplement: Attachment — Submitted filename: Response to Reviewers.docx [file pone.0258950.s005.docx]

**Reviewer #1:** Main findings of the study:

The work reports the characterization, namely in terms of cell viability and gene expression profiles, of cells dispersed by P. aeruginosa biofilms upon treatment with tobramycin (TOB) alone and in combination with alginate lyase (AlyP1400). First, the authors used flow cytometry to estimate the biofilm released cells and to discriminate them into live and dead. Second, transcriptomics was performed in released biofilm cells, where the expression of 12 genes related to virulence or resistance was analyzed. Afterwards (although not mentioned in the abstract), the survival ability of biofilm released cells to a subsequent TOB treatment was addressed by plating the biofilm dispersal cells onto LB agar medium supplemented with TOB at sub-MICs. This is a continuing study of a previous work where the authors demonstrated a reduction in the biomass and degradation of alginate within the EPS matrix of P. aeruginosa biofilms, thus enhancing the antibiofilm activity of TOB.

The findings in this present study showed that TOB+AlyP1400 could increase the number of biofilm-released cells, which were apparently stained green (live) in their majority. At the gene expression level, these cells (when treated with TOB+AlyP1400) had upregulated 4 out of 12 virulence- or resistance-associated genes (bdlA, mexF, mexY, ndvB). Survival studies displayed a significant high TOB tolerant fraction of cells (60%) upon treatment with TOB+AlyP1400.


Limitations and strengths:
Although the work does not fall into an original topic, the paper shows relevant results regarding the use of combinatorial treatment employing biofilm disruptive agents and conventional antibiotics. There are, in fact, multiple studies reporting synergistic activity of alginate lyase with many antibiotic agents, including TOB. However, this study focuses on the characterization of biofilm-released cells upon treatment with alginate lyase+TOB. These cells should not be overlooked and the results sounds the alarm for potential likelihood of severe reinfections by individual cells treated with this therapeutic approach. Overall work and its goal are logically presented. The introduction defines clearly the rationale of the work, considering the fact that the authors have already reported the potentiality of alginate lyase against P. aeruginosa biofilm biomass and its ability to boost TOB action towards these infections.

The methods used in the work are generally well detailed and can be reproduced by others. The results and data interpretation are clearly presented.

Nevertheless, in some parts, it becomes difficult to read and follow. The paper contains some errors in the scientific terminology and should be carefully reviewed and rewritten in some parts.

**There are several issues that need to be addressed before publication:**

1. Alginate formation by *Pseudomonas aeruginosa* is an important bacterial resistance mechanism, by completely preventing or blocking the diffusion of various antibiotics, namely aminoglycosides. While applying alginate lyase, this will disrupt biofilm matrix and allow TOB to penetrate and target biofilm-encased cells. I am wondering if the dispersal of biofilm cells (which are mostly live in these study) happens immediately after biofilm disruption by alginate lyase or the cells will be slowly being dispersed to the environmental milieu… It would be interesting to evaluate also co-administration of AlyP1400 followed by TOB and compare the results with that obtained in this study. Moreover, what happens to the cells residing in the biofilm? Are they live or dead?
**RE: The point that the reviewer raised about the timing when the dispersal of biofilm cells happened after the treatment is very compelling. The data in our original submission showing that the dispersal rates were highest at time zero after the treatment once the flow resumed (Figure 1 & supplementary Figure 2, text between lines 212-215) suggested an immediate rather than gradual dispersal was more likely.**

**In addition, we have taken the reviewer’s suggestion of incorporating the condition of sequential treatment of AlyP1400 followed by tobramycin in the dispersal assay. As shown in the new data included in Figure 1 of the revised submission, the sequential treatment did not cause a difference in dispersal of live cells measured by fluorescence and colony forming unit (Figure 1 A & B) than the non-treated or tobramycin-treated biofilms. The sequential treatment also led to the same dispersal of dead cells with the non-treated biofilm. Because the tobramycin was added to the biofilms in the flow cell after the initial AlyP1400 treatment and incubated for an additional 2 hours before the flow resumed (details in the Method section), the fact that there was no enhanced biofilm dispersal after the initial 2-hour treatment of AlyP1400 suggested that the increased dispersal cells caused by the AlyP1400 treatment is likely to happen immediately after biofilm disruption rather than gradually after the disruption and is not long lasting. This is consistent with the observation on the peak dispersal at time point zero after the resumption of the flow.**

**For the viability of the cells residing in the biofilm after treatment, the viability staining data are shown in Supplementary Figure 1. The quantification of the viability was published in our previous manuscript (references 15&16 in this submission). The focus of the current manuscript is on dispersal cells, therefore the Supplementary Figure 1 showing the viability of remaining biofilm cells was only included to aid in the coherent understanding of the overall effects of our treatments on biofilm.**

2. In this work, the authors used a mucoid CF isolated strain (CF27) to grow biofilms for 96 h, which were then treated (with TOB alone or TOB+AlyP1400) and further tested on their viability, genetic expression and survival upon a second TOB single treatment. However, I am concerned whether this is representative of a major population of isolated strains (i.e. if other CF isolate were used, their behavior would be similar to this one of the CF27? Moreover, why do not include a reference strain, maybe P. aeruginosa ATCC 27853, because it is well known that isolates may have resistance-associated backgrounds that may interfere with later results?
**RE: We have included a lab references strain of *P. aeruginosa* PAK. Not surprisingly, the combinational treatment of tobramycin plus AlyP1400 did not enhance live cell dispersal from biofilms of PAK, a non-mucoid lab strain (Fig. S3), since other exopolysaccharides, but not alginate, are the major components of the biofilm matrix of PAK. We have also tested the strain PAK in the tobramycin tolerant assay and showed that non-treated and treated dispersal cells all had tolarent fractions lower than 3%. These data strongly suggest that the observed tolerance enhancement very likely to be dependent on the specific resistance-associated genetic background of different isolates.**

3. Concerning the age of the biofilm, why the authors have selected 96h to have old mature biofilms? What is the advantage of using biofilms at 96 h instead of 24 or even 48h?

**RE: The cultivation of *P. aeruginosa* biofilm in a flow cell continuous system was optimized and described in our previous studies (references 15&16 in this submission).** **The rationale for choosing the 96-h old mature biofilms over the 24h- or 48h-old ones is that the stability and consistency of the 96-h ones are superior to the others. This point has been included in the first section “*P. aeruginosa* biofilm cultivation and treatments” in the Materials and Methods.**

4. Why forming biofilms on flow dynamic cell systems having glass as substratum? What is the advantage of biofilms formed on these devices and biofilms formed on reproducible microtiter plates, for example?
**RE: The justification is similar to the point above. The advantage of forming biofilms on flow dynamic cell systems having glass as substratum was provided in our previous publications. That is, it was a robust method for mimicking the respiratory tract of CF patients. In addition, the flow system enables easy and consistent collection of the dispersal cells at different time points post treatment, which are the targets of investigation of this work. This point has also been included in the first section “*P. aeruginosa* biofilm cultivation and treatments” in the Materials and Methods.**

5. If not previously addressed, it would be interesting to evaluate the effect of the combination of the alginate lyase and TOB through the FIC outcome (checkerboard assay) against *P. aeruginosa*.
**RE: A great point. These data have been published in our previous work (reference 16 in this manuscript). It was shown using the checkerboard assay that the combination of the alginate lyase and TOB was able to decrease the MBEC against *P. aeruginosa* biofilm but not MIC against planktonic cells, which is consistent with our proposed mode of action for alginate lyase.**

6. I think having a schematic representation of the concept of the study would be valuable.
**RE: We have included a schematic model figure in the revision.**

**Reviewer #2:** The manuscript by Daboor et al., aims to examine the viability and phenotype of chemically-induced dispersal of cells from Pseudomonas aeruginosa biofilms. Using a previously characterized clinical isolate, the authors confirm previous dispersal results and attempt to quantify the viable cellular material using fluorescent markers for viability. Next they studied the expression of a limited number of genes related to antibiotic resistance and virulence. Their findings indicate that some of these genes are increased in expression when cells are exposed to antimicrobials and dispersal agents.

The overall goal of the manuscript, which is to study the mechanics of anti-alg based treatments in conjunction with tobramycin are appealing. There is a lack of work in the determining the mechanisms and more importantly the phenotypes related to the process of biofilm dispersal. However, the authors results are do not convincingly make the case that they are actually, or accurately detecting this process. Much technical insight/information and data are required to support their observations, specifically with respect to the how dispersal is quantified. Below, several major areas that require addressing are discussed. The gene expression data is intriguing and are the highlight of the paper, but on their own seem superficial and not well supported.


Comments:

- The description of how displeased cells were measured relative to the initial biofilm biomass is poorly described. How consistent were each biofilm in prior to treatment in terms of number of cells (CFU/ml) and does the variability affect the dispersal over time?
**RE: We agree that a consistent initial biofilm biomass is key to have meaningful comparison of the effectiveness of the combinational treatment. We have optimized the biofilm cultivation conditions in our previous work. Based on our previously published data (references 15 & 16 in this manuscript), each biofilm at 96-h after incubation and in prior to treatment, in terms of biomass measured by crystal violate staining on 96-well ELISA plates, as well as fluorescence intensities within the flow cells, are highly consistent and stable. The description of this normalization to uniform initial biofilm has been included in the first section “*P. aeruginosa* biofilm cultivation and treatments” in the Materials and Methods.**

- Starting at line 212, the authors state that maximal dispersal occurs at zero hours - how was this determined and where is it described in Figure 1? Can the data be presented so that the time dependence is clearly discernable? The A, B, C designation to indicate the level of error in Figure 1 is also confusing as the panels are labelled similarly.
**RE: To facilitate the straightforward comparison of different treatments and to avoid overcrowding the figure with numerous parameters, we presented the data points at the zero hour (Figure 1) separately from the rest of the time points (Supplementary Figure 2) in our original submission. We agree that a direct comparison between different time points will help the clarity of the data. We have included the data for time zero in both Figure 1 as a standalone figure to focus on the comparisons of different treatments, as well as in Supplementary Figure 2 together with the other time points.**

**The labels for statistical analysis result were changed to lower letters in order to avoid the confusion with the similarity with the panel labels.**

In Figure 1D, the differences observed with the with the combination treatment of AlyP1400+Tob are not convincingly different than non-treated or Tob treated cells.

**RE: Although the absolute quantities of the dispersal caused by the combinational treatment were significantly higher than the non-treated and tobramycin-treated biofilms as shown in Figure 1 panels A-C, the ratios of dead/live cells under all conditions were indeed not different with each other as the reviewer pointed out. The writing of the result for that part was revised to clarify this.**
